# Ophthalmology

**Published:** 1904-10-01

**Authors:** 


### OPHTHALMOLOGY.

**Eye-strain as a Cause of Headache and Other Neuroses.**—Snell,[1] in a very seasonable paper on the above subject points out how small an amount of cylindrical error may produce headaches and the other troubles that follow eye-strain. He quotes from 800 cases and says, there is in some quarters a failure to recognise the importance of estimating these low degrees of astigmatism and the value derived from the use of weak cylinders. A careful study of the subject, however, will easily demonstrate that low degrees of astigmatism play a very important part in the causation of the neuroses under consideration. They will be found in patients complaining little, if at all, of their sight, and can only be detected and the exact axis ascertained after a careful examination, when the eye is under the action of a mydriatic. He refers especially to astigmatism of 0.25 D. and 0.5 D. In his series of 144 cases of hypermetropic astigmatism, simple and compound, he finds that +.25 D. cylinder was prescribed in 18 cases of simple and five of compound hypermetropic astigmatism; +.50 D. cylinder in 38 and nine respectively; and +.75 D. cylinder in 22 and eight respectively, thus making a total of 100 out of the 144 cases in which cylinders of +.75 D. and under were prescribed. The benefit of these weak plus cylinders was undoubted. There were also 34 cases of myopic astigmatism. For these cylinders of —.75 D. and lower were ordered in 20 cases, viz. —0.25 D. cylinder in four cases, —0.5 D. cylinder in six cases, and —.75 D. cylinder in 10 cases. The percentage of these weak cylinders works out as 66.8 per cent. for hypermetropic and 58 per cent. for myopic astigmatism. We are rather surprised to read this, as we usually considered that although hypermetropic astigmatism, even of low degree, caused much headache and eye worry, myopic astigmatism of low degree caused very little trouble, and to this conclusion we were led from personal experience in the matter. The American ophthalmic surgeons have used cylinders + and — .25 D. for a long time, but it was thought that this was necessary only because the Americans are such highly neurotic people, that very small amounts of ametropia are sufficient to cause them considerable trouble. Snell summarises his paper as follows:—1. That eye-strain is the cause of a large proportion of headaches, often of a very aggravated character. 2. That various other neuroses are met with in association with headache, and among these may be mentioned: mental depression, nausea, indigestion, vomiting, insomnia, giddiness, choreiform movements of the eyelids and face. 3. That relief is afforded to these conditions by correcting the error of refraction, which can be ascertained only after careful examination. 4. That for such examination a mydriatic is absolutely essential. 5. That frequently no complaint is made of defect of vision. 6. That the ametropia is frequently of low degree. 7. That a cylinder of 0.25 D. is of great value. 8. That anisometropia is frequently present and requires proper adjustment. 9. That in a certain number of cases the muscle balance is faulty and necessitates the prescribing of prisms. While on this subject one might refer to a great discussion, a kind of "battle of the giants," which has recently been carried on, owing to an article, or rather series of articles, published by Gould of Philadelphia, showing, according to him, that what were called eccentricities of genius were no doubt due to this eye-strain, caused doubtless by astigmatism, which was uncorrected, for in those days astigmatism was not recognised as a cause of headache and eye worry. The discussion led to several rather acrimonious letters on both sides, and as] these discussions generally do, has led to but little result, each side marching off with flying colours and thinking that they have won the day. We should think the trouble lay between the two and that each was a little right and a little wrong. There can be no doubt that astigmatism of low degree does cause worry in some cases, but "all the ills that flesh is heir to" cannot be put down to astigmatism. Some time ago an American ophthalmic surgeon published a book showing clearly, according to his lights, that all the human frailties including dysmenorrhœa, and such like were due to a faulty muscle balance of the eyes, and practically that by cutting the various eye muscles judiciously they could be easily cured; this work was treated on this side of the water with due reserve and the history of the results taken with a very large grain of salt and there the matter ended.

**Choroidal Inflammation with Loss of Vision Caused by Excessive Use of the Eyes for a Short Period.**—St. John Roosa [2] quotes a very interesting case of a physician, 46 years old, who, while attending a confinement case, read a book almost continuously for 10 hours. The print was small, but not excessively so. He was confident that his vision up to that period was excellent in each eye. Next morning his vision was blurred at first in both eyes, but afterwards only in the right one. The blur was very disturbing, and after six weeks' worry he sought advice. The vision was then normal in the left eye, while that of the right was reduced to 5/9, the vitreous humour being hazy, so that a clear view could not be obtained of his right fundus. He was not seen again for two years, and then the vision was just the same, but the haziness of the vitreous had cleared up, and it was then seen that there were several spots of choroidal atrophy over which the retinal vessels passed. In 1903, that is seven years after he was first seen, the vision remained the same, the ophthalmoscope showed the same extensive choroidal changes in the right eye. The left fundus was normal. Roosa comments on the case as follows:—Although continuous use of the eyes on small objects is constantly enumerated in text-books as one of the causes of inflammatory action in the eyeball, such a result is seldom seen by the oculist. One of the reasons may be that the asthenopia, which generally follows excessive use, usually causes the patient to desist from undue occupation with his eyes before the danger-line has been crossed. The relation of cause to effect in the case he reports is pretty distinct. The observer was intelligent, had good occasion to watch his vision in his work, and states distinctly that he never felt the blur till the morning after this continued use. Probably the choroiditis was set up at that time. It is also remarkable that the disease came to an immediate standstill, so far as impairing the function of vision is concerned. One would have expected the choroiditis would advance, as the patient took no extraordinary means to protect his eyes from this danger. It is interesting to note that the retina has not been invaded—to any very considerable degree, at least—by this inflammatory action. The process attacked the vascular part of the eyeball, and immediately invaded the vitreous, causing the hyalitis, which was all there was to be seen at the first examination. It is also interesting to note that this is a traumatism rather than an infection of the eye. In later times, with our advanced knowledge of infectious diseases, we sometimes are inclined to ignore primary, mechanical, and chemical causes, which apparently produce conditions entirely similar to those from pure infection.

**Spring Catarrh.**—Dr. Posey,[3] of Philadelphia, lately sent a circular letter to the ophthalmologists of the United States with a view to ascertaining their experiences with reference to spring catarrh. Their answer showed that it occurred in the proportion of 1 to every 200 or 300 cases of conjunctival disease, and that it is not increasing in frequency. The palpebral type is most common (60 per cent.), the associated form comes next (30 per cent.), and the ocular form is least common (10 per cent). The elevations in the conjunctiva are more common than the thickening of the conjunctiva; both lids, as a rule, are affected. In about 7 per cent. the disease is limited to one eye. The age limits are from 18 months to 50 years. Males are more frequently affected (85 per cent.) than females (15 per cent.). It is questionable whether there is any connection between vernal conjunctivitis and diseases of the nose and throat. It is not considered to be contagious, but it may sometimes be hereditary. Treacher Collins says that he saw a gentleman who had consulted him for this troublesome complaint, and who had consulted 10 well-known oculists of whom five told him he had trachoma and the other five that he had spring catarrh. It shows how difficult the diagnosis is between these two affections clinically. The histological appearances, however, are quite distinct. In trachoma there would certainly be nodules of lymphoid tissue present, while in spring catarrh there would be large flat-topped papillæ composed of fibrous tissue and no lymphoid follicles. With regard to the treatment we again quote Dr. Posey's results: Nearly everything in the ophthalmic materia medica has been tried, as well as operative procedures, but weak astringent washes with occasional massage with yellow oxide of mercury ointment, give the best results. Adrenalin is found to relieve the intense itching, and iced compresses and lotions are also useful. From all this we may surmise that even after the diagnosis has been made the treatment is not very satisfactory.

[1] Lancet, April 30, 1904. [2] Med. Rec., Feb. 20, 1904. [3] Practioner, March 1904.

